# Identifying Liver Cancer-Related Enhancer SNPs by Integrating GWAS and Histone Modification ChIP-seq Data

**DOI:** 10.1155/2016/2395341

**Published:** 2016-06-27

**Authors:** Tianjiao Zhang, Yang Hu, Xiaoliang Wu, Rui Ma, Qinghua Jiang, Yadong Wang

**Affiliations:** ^1^School of Computer Science and Technology, Harbin Institute of Technology, Harbin 150001, China; ^2^School of Life Science and Technology, Harbin Institute of Technology, Harbin 150001, China

## Abstract

Many disease-related single nucleotide polymorphisms (SNPs) have been inferred from genome-wide association studies (GWAS) in recent years. Numerous studies have shown that some SNPs located in protein-coding regions are associated with numerous diseases by affecting gene expression. However, in noncoding regions, the mechanism of how SNPs contribute to disease susceptibility remains unclear. Enhancer elements are functional segments of DNA located in noncoding regions that play an important role in regulating gene expression. The SNPs located in enhancer elements may affect gene expression and lead to disease. We presented a method for identifying liver cancer-related enhancer SNPs through integrating GWAS and histone modification ChIP-seq data. We identified 22 liver cancer-related enhancer SNPs, 9 of which were regulatory SNPs involved in distal transcriptional regulation. The results highlight that these enhancer SNPs may play important roles in liver cancer.

## 1. Introduction

Single nucleotide polymorphism (SNP) is a variation at a single nucleotide in a DNA sequence [1]. In the last decade, a large number of genome-wide association studies (GWAS) have been published, indicating that thousands of SNPs are associated with diseases. Linkage disequilibrium is the nonrandom association of alleles at different genome locations [2]. There are many SNPs in LD with the causal SNP at specific GWAS locus [3, 4]. Over 90% of these GWAS variants are located in noncoding regions, and approximately 10% are in LD with a protein-coding variant [5, 6]. In protein-coding regions, many studies have shown that some SNPs are associated with numerous diseases by affecting gene expression [7, 8]. However, in noncoding regions, the mechanism of how SNPs contribute to disease susceptibility remains unclear.

Enhancers are the core regulatory components of the genome that act over a distance to positively regulate gene expression [9]. It is estimated that 400,000 to 1 million putative enhancers exist in the human genome [10, 11]. Recently, some studies have shown that disease-related GWAS SNPs are correlated with enhancers marked with special histone modifications [12–15]. Therefore, through integrating GWAS and histone modification ChIP-seq data in a given disorder, we can identify disease-related enhancer SNPs.

We provided a method for identifying liver cancer-related enhancer SNPs through integrating liver cancer GWAS and histone modification ChIP-seq data. We identified 22 enhancer SNPs associated with liver cancer, 9 of which were regulatory SNPs involved in distal transcriptional regulation. The results highlight that these enhancer SNPs may play important roles in liver cancer.

## 2. Results

### 2.1. Pipeline of Identifying Liver Cancer-Related Enhancer SNPs

**Figure 1:**
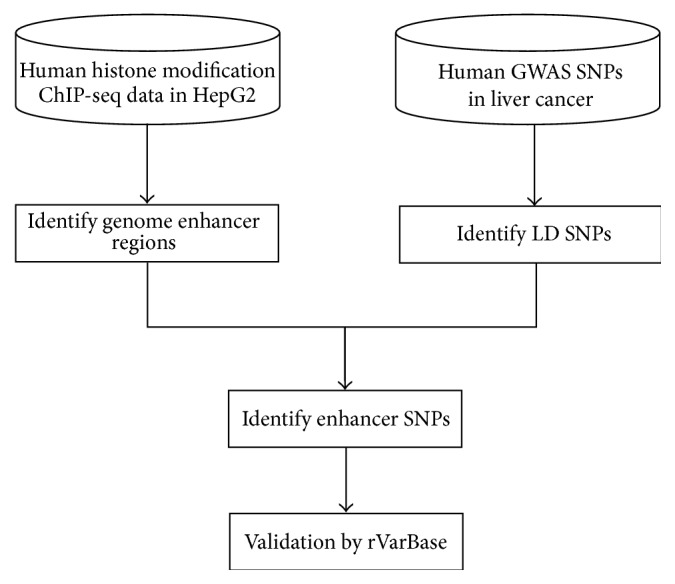
Flowchart identifying liver cancer-related enhancer SNPs. LD SNPs: SNPs located in linkage disequilibrium blocks of GWAS SNPs. Enhancer SNPs: SNPs identified in enhancer regions. rVarBase: a database for variant's regulatory features.

As shown in Figure 1, the pipeline consists of four steps. Firstly, we downloaded liver cancer-related GWAS SNPs from the GRASP [16] database and used LD data from HapMap [17] to infer liver cancer-related LD SNPs. Secondly, we identified enhancer regions in liver cancer through integrating histone modification ChIP-seq data in the HepG2 cell line. Thirdly, we mapped the liver cancer-related LD SNPs to the identified enhancers in liver cancer and obtained liver cancer-related enhancer SNPs. Finally, we used a curated regulatory SNP database named rVarBase [18] to validate our results.

### 2.2. Linkage Disequilibrium Analysis with Liver Cancer-Associated SNPs

We obtained 45 liver cancer-associated SNPs from GRASP (Table 1). These SNPs are the raw potential liver cancer-related SNPs. Then, we used LD data from HapMap to achieve liver cancer-associated LD SNPs. The total number of potential liver cancer-related SNPs is 340.

### 2.3. Identification of Liver Cancer-Related Enhancer SNPs

Previous studies indicated that the enhancer regions are marked by a strong H3K4me1 signal and a relatively weak H3K4me3 signal [19, 20]. Thus, we used histone modification ChIP-seq data to recognize the enhancer regions in liver cancer. Then, we mapped the liver cancer-related GWAS SNPs to the enhancer regions and obtained 22 enhancer SNPs in liver cancer (Table 2).

### 2.4. Validation as Regulatory SNPs

rVarBase is a database that provides reliable, comprehensive, and user-friendly annotations on variant's regulatory features [18]. It includes regulatory SNPs (rSNPs), LD-proxies of rSNPs, and genes that are potentially regulated by rSNPs. We used rVarBase to analyze these 22 enhancer SNPs in liver cancer and found that 14 SNPs have evidence of regulatory SNPs and 9 SNPs (rs9494257, rs6903949, rs6996881, rs4739519, rs6988263, rs12156293, rs1568658, rs5994449, and rs5753816) are involved in distal transcriptional regulation (Table 3).  Table 4 shows the potential target genes of these 9 SNPs.

## 3. Materials and Methods

### 3.1. GWAS and LD Datasets

We downloaded the human liver cancer-related GWAS SNPs from GRASP. The database includes 26 and 19 liver cancer-associated SNPs (*p* < 10^−5^) from Han Chinese in Beijing, China (CHB), and Japanese in Tokyo, Japan (JPT), respectively. The URL is https://grasp.nhlbi.nih.gov/Overview.aspx. We obtained all SNPs in LD with GWAS-lead SNPs using LD blocks identified with publicly available HapMap data on the CHB and JPT populations. The LD data can be downloaded from http://hapmap.ncbi.nlm.nih.gov/index.html/.

### 3.2. Histone Modification Datasets

We downloaded the human histone modification ChIP-seq datasets in the HepG2 cell line from the ENCODE Production Data/Broad Institute. The URL is http://genome.ucsc.edu/ENCODE/downloads.html.

### 3.3. Linkage Disequilibrium Analysis

In the genome, SNPs located in close proximity tend to be in linkage disequilibrium with each other. The International HapMap Project has established linkage disequilibrium of human genome SNPs. We used LD data from HapMap to achieve liver cancer-associated LD SNPs (*R* ^2^ > 0.8).

### 3.4. Identify Enhancer Regions and Enhancer SNPs

Firstly, we downloaded the human histone modification BAM files (H3K4me1 and H3K4me3) in the HepG2 cell line from the ENCODE project. Then, we used BEDtools [21] to count read coverage for every position of the genome. Through calculating the ratio H3K4me1/H3K4me3 and picking up the regions with log_2_(H3K4me1/H3K4me3) > 1.2, we identified the potential enhancer regions. Finally, we mapped the potential LD SNPs to these enhancer regions and achieved liver cancer-related enhancer SNPs.

## 4. Discussion

Through integrating liver cancer GWAS SNPs from GRASP, LD data from HapMap, and histone modification ChIP-seq data from ENCODE, we explored liver cancer-related enhancer SNPs. We compared our results with rVarBase and found that 9 SNPs (rs9494257, rs6903949, rs6996881, rs4739519, rs6988263, rs12156293, rs1568658, rs5994449, and rs5753816) were regulatory SNPs involved in distal transcriptional regulation. The results highlight that these enhancer SNPs may play important roles in liver cancer.

Compared with protein-coding regions in the human genome, noncoding regions contain much more genetic variations. Some important regulation regions, such as enhancers, have great influence on target gene expression. SNPs located in these regions may disturb gene expression and even cause diseases. Thus, the identification of SNPs in enhancer regions is helpful to understand the mechanism of association between SNPs and diseases.

We presented a method to identify disease-related SNPs located in enhancer regions that gives a new solution to investigate the relationship between SNPs and diseases. The presented method can also be applied to other diseases and will enable biologists to investigate the mechanism of disease risk associated with SNPs.

## Figures and Tables

**Table 1:** Summary of liver cancer-associated SNPs from GRASP database.

SNP ID	*p* value	Chromosome	Populations	PMID
rs17401966	1.20*E* − 19	1	CHB	20676096
rs1249458	8.30*E* − 06	2	CHB	22807686
rs1714259	1.10*E* − 06	2	CHB	22807686
rs2396470	5.10*E* − 07	2	CHB	20676096
rs7424161	8.80*E* − 06	2	CHB	22807686
rs7574865	1.70*E* − 11	2	CHB	23242368
rs3905886	3.70*E* − 06	3	CHB	22807686
rs1073547	6.80*E* − 06	4	CHB	22807686
rs17081345	3.70*E* − 07	6	CHB	20676096
rs9272105	3.30*E* − 23	6	CHB	22807686
rs9275319	8.70*E* − 19	6	CHB	23242368
rs9494257	1.10*E* − 14	6	CHB	20676096
rs12682266	6.70*E* − 06	8	CHB	22174901
rs1573266	7.40*E* − 06	8	CHB	22174901
rs2275959	6.40*E* − 06	8	CHB	22174901
rs7821974	7.00*E* − 06	8	CHB	22174901
rs7898005	7.00*E* − 08	10	CHB	20676096
rs10160758	6.00*E* − 06	11	CHB	22807686
rs10896464	6.50*E* − 06	11	CHB	22807686
rs2611145	9.30*E* − 06	11	CHB	22174901
rs3825023	3.10*E* − 06	11	CHB	22807686
rs7119426	3.50*E* − 06	11	CHB	22807686
rs402071	8.60*E* − 06	19	CHB	22807686
rs3092194	4.40*E* − 06	20	CHB	22807686
rs368007	9.90*E* − 06	20	CHB	22807686
rs455804	4.40*E* − 10	21	CHB	22807686
rs1980215	2.30*E* − 06	3	JPT	21499248
rs2596542	4.20*E* − 13	6	JPT	21499248
rs9275572	1.40*E* − 09	6	JPT	21499248
rs1568658	6.90*E* − 06	7	JPT	21499248
rs952656	2.80*E* − 06	8	JPT	21499248
rs4363614	4.20*E* − 07	11	JPT	21499248
rs1957496	4.60*E* − 06	14	JPT	21499248
rs8019534	3.90*E* − 06	14	JPT	21499248
rs1794304	3.60*E* − 06	16	JPT	21725309
rs2208456	3.80*E* − 06	20	JPT	21499248
rs1012068	1.30*E* − 14	22	JPT	21725309
rs11703779	4.20*E* − 06	22	JPT	21725309
rs4820994	3.00*E* − 06	22	JPT	21725309
rs4820996	4.20*E* − 06	22	JPT	21725309
rs5753816	4.90*E* − 06	22	JPT	21725309
rs5753818	9.40*E* − 06	22	JPT	21725309
rs5998152	1.20*E* − 07	22	JPT	21725309
rs7287054	3.80*E* − 06	22	JPT	21725309
rs737084	5.90*E* − 06	22	JPT	21725309

**Table 2:** Summary of predicted enhancer SNPs in liver cancer.

SNP ID	Chromosome	Start	End	Chain	Populations
rs12751375	chr1	10291873	10291874	+	CHB
rs6700866	chr1	10306037	10306038	+	CHB
rs9494257	chr6	135827471	135827472	+	CHB
rs17064474	chr6	135680137	135680138	+	CHB
rs17721919	chr6	135748923	135748924	+	CHB
rs17721931	chr6	135749377	135749378	+	CHB
rs6903949	chr6	135821065	135821066	+	CHB
rs6996881	chr8	37407919	37407920	+	CHB
rs4739519	chr8	37412858	37412859	+	CHB
rs6988263	chr8	37414659	37414660	+	CHB
rs12156293	chr8	37419921	37419922	+	CHB
rs6928810	chr6	31410523	31410524	+	JPT
rs3869132	chr6	31410947	31410948	−	JPT
rs2596562	chr6	31354594	31354595	−	JPT
rs2523475	chr6	31361709	31361710	−	JPT
rs2523467	chr6	31362929	31362930	−	JPT
rs9501387	chr6	31364458	31364459	+	JPT
rs1568658	chr7	29141557	29141558	−	JPT
rs1794304	chr16	12625394	12625395	+	JPT
rs5994449	chr22	32304178	32304179	+	JPT
rs5753816	chr22	32312841	32312842	+	JPT
rs5749339	chr22	32315734	32315735	+	JPT

**Table 3:** Summary of liver cancer-related regulatory SNPs validated by rVarBase.

SNP ID	Regulatory SNP	Distal regulation	Chromatin state	Related regulatory elements
rs12751375	Yes	No	Inactive region	n/a
rs6700866	Yes	No	Weak transcription; ZNF genes and repeats; strong transcription; enhancers	n/a
rs9494257	Yes	Yes	Enhancers; flanking active TSS; weak transcription	Chromatin interactive region
rs17064474	Yes	No	Weak transcription; active TSS; flanking active TSS; enhancers	n/a
rs17721919	Yes	No	Weak transcription	n/a
rs17721931	Yes	No	Weak transcription	n/a
rs6903949	Yes	Yes	Weak transcription; enhancers	TF binding region; chromatin interactive region
rs6996881	Yes	Yes	Weak transcription; enhancers	Chromatin interactive region
rs4739519	Yes	Yes	Enhancers; weak transcription	Chromatin interactive region
rs6988263	Yes	Yes	Enhancers; weak transcription; genic enhancers; bivalent enhancer; flanking active TSS	Chromatin interactive region
rs12156293	Yes	Yes	Enhancers; weak transcription; bivalent enhancer; genic enhancers	Chromatin interactive region
rs1568658	Yes	Yes	Weak transcription; enhancers; strong transcription	Chromatin interactive region
rs5994449	Yes	Yes	Weak transcription; strong transcription; ZNF genes and repeats	Chromatin interactive region
rs5753816	Yes	Yes	Weak transcription; enhancers; flanking active TSS	Chromatin interactive region

**Table 4:** Summary of liver cancer-related regulatory SNPs and potential target genes validated by rVarBase.

SNP ID	Gene symbol	Ensemble ID	Regulation type
rs9494257	BCLAF1	ENSG00000029363	Distal transcriptional regulation
rs9494257	AHI1	ENSG00000135541	Distal transcriptional regulation
rs9494257	LINC00271	ENSG00000231028	Distal transcriptional regulation
rs6903949	MYB	ENSG00000118513	Distal transcriptional regulation
rs6903949	BCLAF1	ENSG00000029363	Distal transcriptional regulation
rs6903949	AHI1	ENSG00000135541	Distal transcriptional regulation
rs6903949	LINC00271	ENSG00000231028	Distal transcriptional regulation
rs6996881	ZNF703	ENSG00000183779	Distal transcriptional regulation
rs6996881	ERLIN2	ENSG00000147475	Distal transcriptional regulation
rs6996881	Null	ENSG00000183154	Distal transcriptional regulation
rs6996881	Null	ENSG00000253161	Distal transcriptional regulation
rs4739519	ZNF703	ENSG00000183779	Distal transcriptional regulation
rs4739519	Null	ENSG00000254290	Distal transcriptional regulation
rs6988263	ZNF703	ENSG00000183779	Distal transcriptional regulation
rs6988263	Null	ENSG00000254290	Distal transcriptional regulation
rs12156293	ZNF703	ENSG00000183779	Distal transcriptional regulation
rs12156293	Null	ENSG00000254290	Distal transcriptional regulation
rs12156293	ERLIN2	ENSG00000147475	Distal transcriptional regulation
rs12156293	Null	ENSG00000183154	Distal transcriptional regulation
rs1568658	Null	ENSG00000228421	Distal transcriptional regulation
rs1568658	TRIL	ENSG00000176734	Distal transcriptional regulation
rs1568658	Null	ENSG00000255690	Distal transcriptional regulation
rs5994449	DEPDC5	ENSG00000100150	Distal transcriptional regulation
rs5994449	FBXO7	ENSG00000100225	Distal transcriptional regulation
rs5994449	SYN3	ENSG00000185666	Distal transcriptional regulation
rs5994449	PRR14L	ENSG00000183530	Distal transcriptional regulation
rs5994449	PISD	ENSG00000241878	Distal transcriptional regulation
rs5994449	EIF4ENIF1	ENSG00000184708	Distal transcriptional regulation
rs5994449	RNU6-28	ENSG00000199248	Distal transcriptional regulation
rs5994449	SFI1	ENSG00000198089	Distal transcriptional regulation
rs5753816	YWHAH	ENSG00000128245	Distal transcriptional regulation
rs5753816	C22orf24	ENSG00000128254	Distal transcriptional regulation
rs5753816	PISD	ENSG00000241878	Distal transcriptional regulation
rs5753816	DEPDC5	ENSG00000100150	Distal transcriptional regulation
rs5753816	RNU6-28	ENSG00000199248	Distal transcriptional regulation
rs5753816	SFI1	ENSG00000198089	Distal transcriptional regulation
rs5753816	EIF4ENIF1	ENSG00000184708	Distal transcriptional regulation
rs5753816	RFPL3S	ENSG00000205853	Distal transcriptional regulation
rs5753816	Null	ENSG00000230736	Distal transcriptional regulation
rs5753816	Null	ENSG00000243519	Distal transcriptional regulation
rs5753816	Null	ENSG00000241954	Distal transcriptional regulation
rs5753816	Null	ENSG00000232218	Distal transcriptional regulation
rs5753816	SYN3	ENSG00000185666	Distal transcriptional regulation
